# Supplementary material for: No Evidence of Interaction Between FADS2 Genotype and Breastfeeding on Cognitive or Other Traits in the UK Biobank
Source: Behav Genet. 2024 Dec 9;55(2):86–102. doi: 10.1007/s10519-024-10210-0 (PMC11882634; doi:10.1007/s10519-024-10210-0)
Supplement: Supplementary file 1 — Supplementary file1 (DOCX 28 KB) [file 10519_2024_10210_MOESM1_ESM.docx]

## Supplementary Figure 1: Results from the association analysis between the three FADS2 SNPs (additive effect) and cognitive traits stratified by breastfeeding status


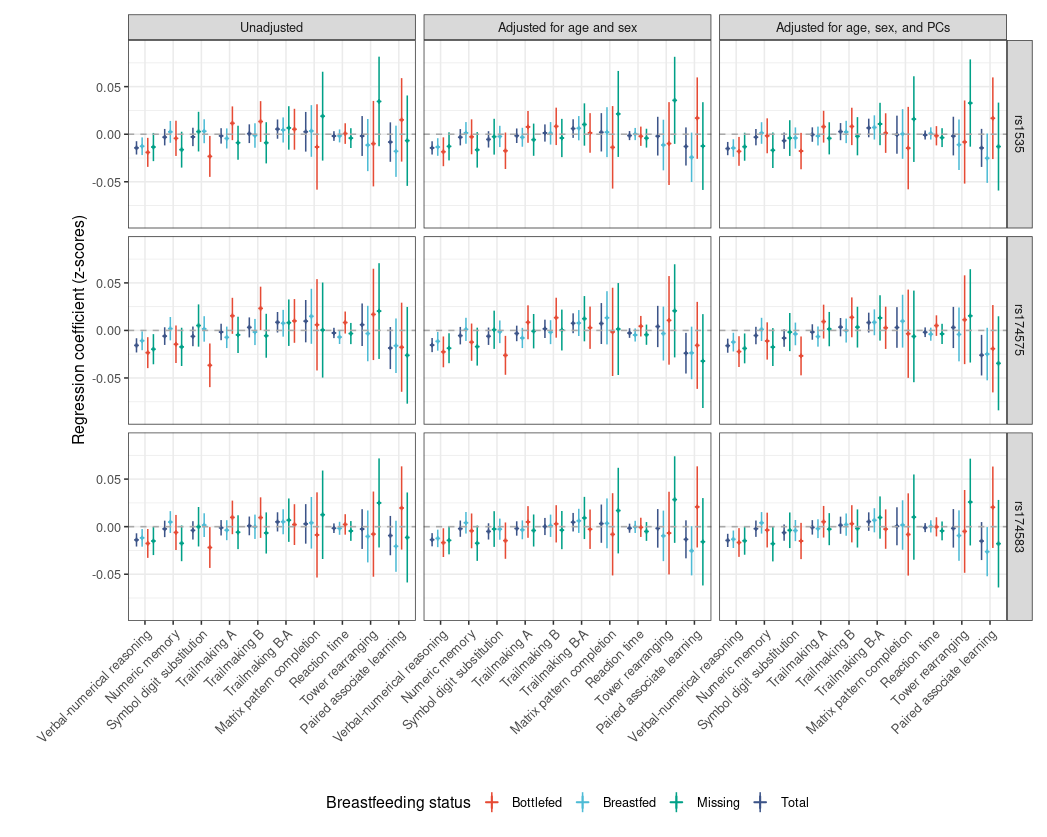


The results are expressed in change of standard deviations of the phenotype per increase in effect allele copies in each SNP. Error bars represent 95% confidence intervals.

## Supplementary Figure 2 Results from the association analysis between the three FADS2 SNPs (additive effect) and noncognitive continuous traits stratified by breastfeeding status for all adjustment strategies
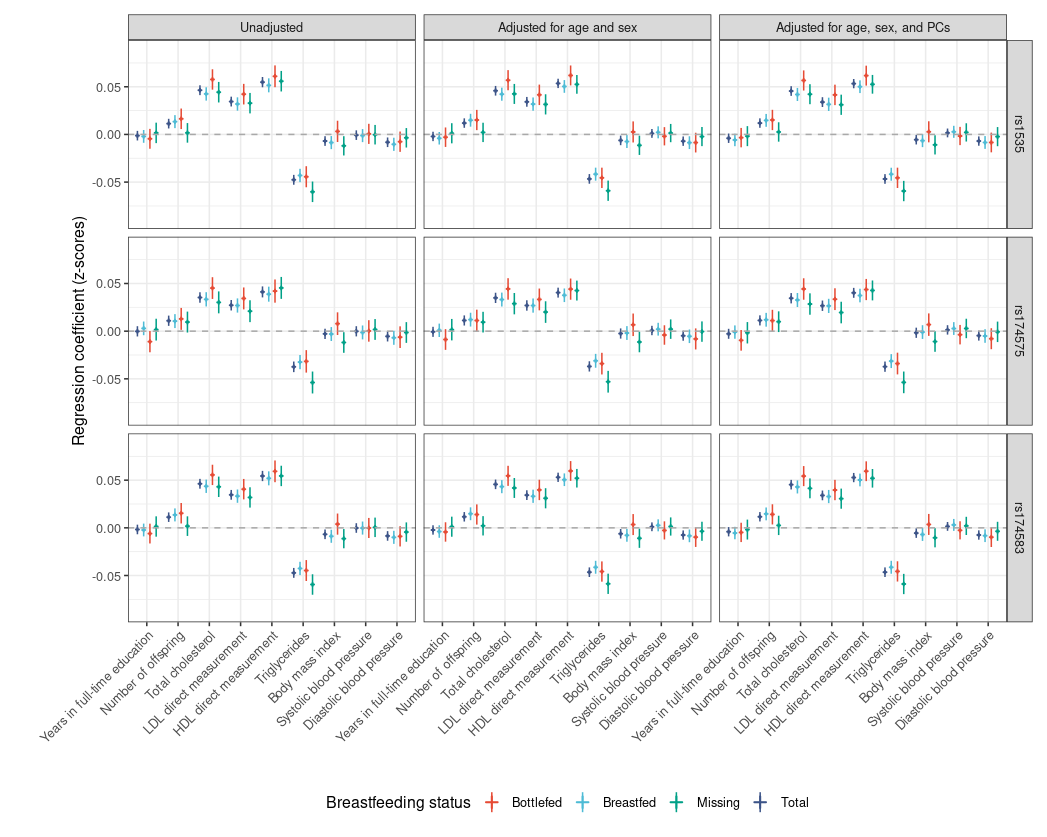


The results are expressed in change of standard deviations of the phenotype per increase in effect allele copies in each SNP. Error bars represent 95% confidence intervals.

## Supplementary Figure 3: Results from the association analysis between the three FADS2 SNPs (additive effect) and binary traits (affected-unaffected) stratified by breastfeeding status for all adjustment strategies
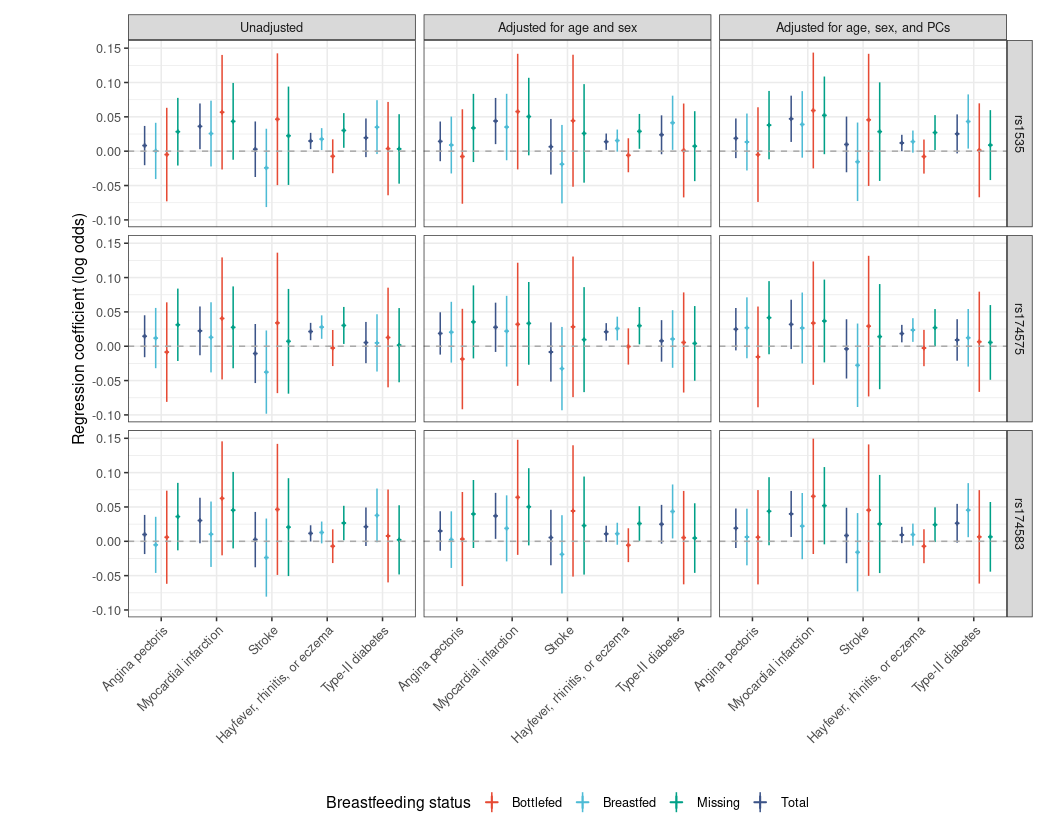


The results are expressed in change of natural log odds of being affected by the phenotype per increase in effect allele copies in each SNP. Error bars represent 95% confidence intervals.

## Supplementary Figure 4: Results from the association analysis between the three FADS2 SNPs (additive effect) and cognitive traits stratified by breastfeeding status for all adjustment strategies, all ancestries


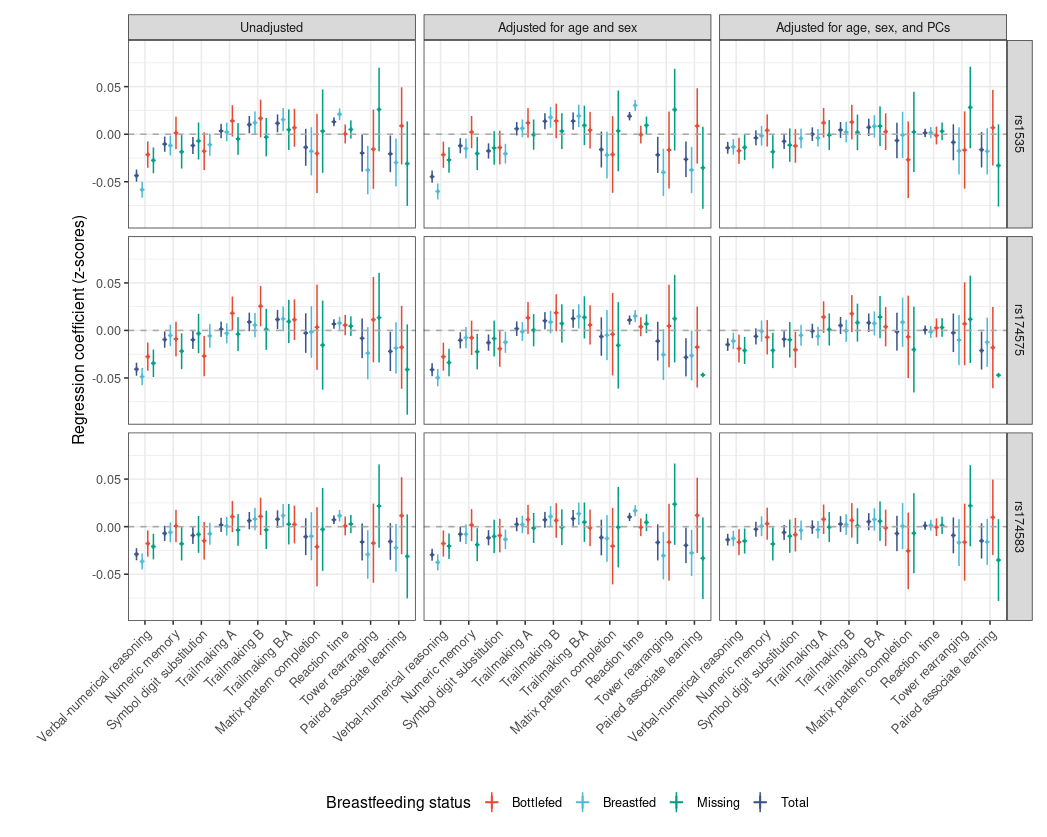


The results are expressed in change of standard deviations of the phenotype per increase in effect allele copies. Error bars represent 95% confidence intervals.

## Supplementary Figure 5: local plot of LD structure in the region surrounding FADS2


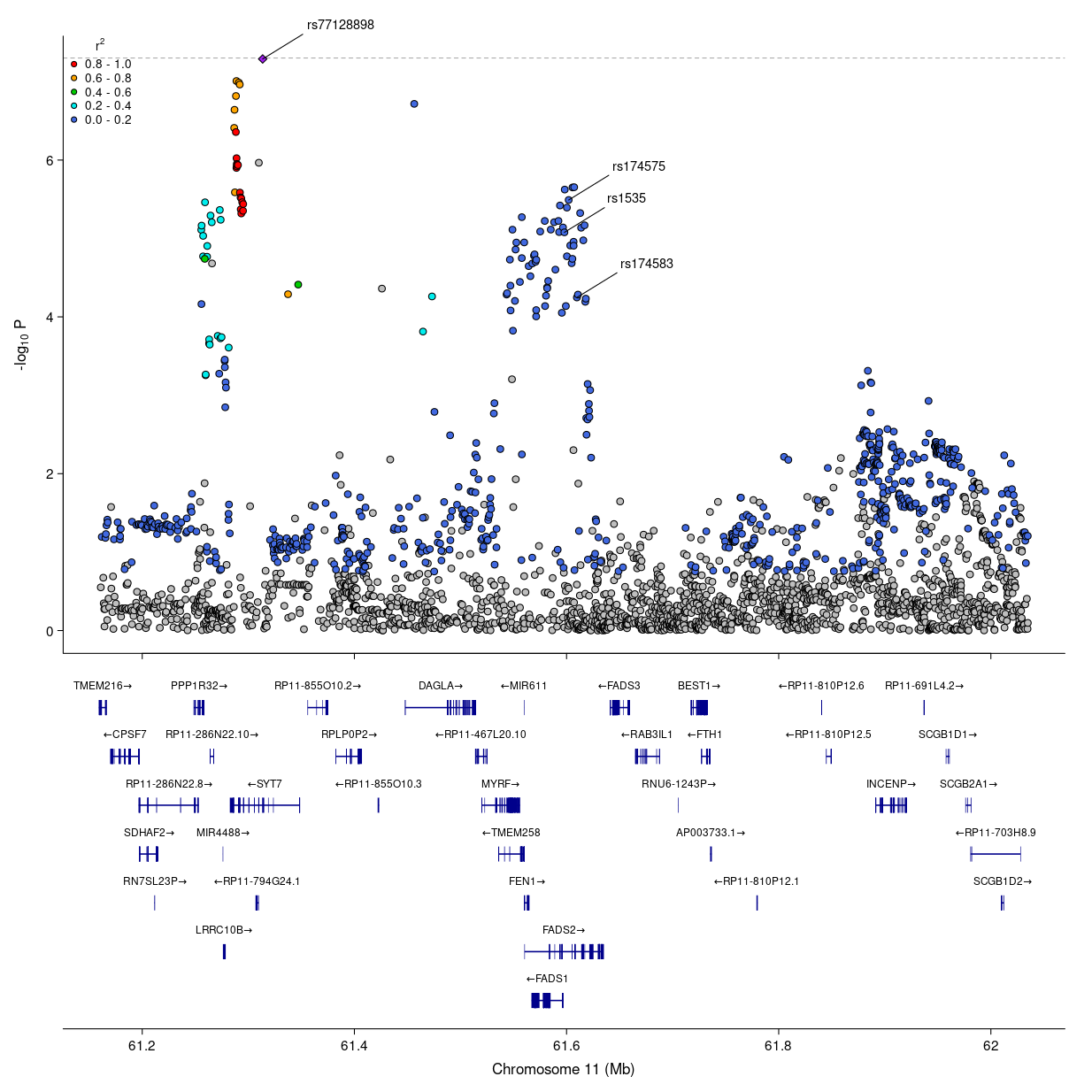


Data from Savage *et al.* (2018). is relative to the nearest top hit (rs77128898) based on the CEU panel (Northern Europeans from Utah) from 1000 Genomes (Auton et al. 2015). Gene tracks were obtained from Ensembl release 75 (Flicek et al. 2014). The dashed line represents the genome-wide significance threshold ($p=5\times{10}^{-8}$).
